# Exercise for Mental Well-Being: Exploring Neurobiological Advances and Intervention Effects in Depression

**DOI:** 10.3390/life13071505

**Published:** 2023-07-04

**Authors:** Jianchang Ren, Haili Xiao

**Affiliations:** Institute of Sport and Health, Guangdong Provincial Kay Laboratory of Development and Education for Special Needs Children, Lingnan Normal University, Zhanjiang 524037, China; xiaohl@lingnan.edu.cn

**Keywords:** exercise, physical activity, neurobiology, depression, antidepressant, inflammation, brain-derived neurotrophic factor, kynurenine, skeletal muscle, aerobic exercise

## Abstract

Depression is a common mental disorder in which patients often experience feelings of sadness, fatigue, loss of interest, and pleasure. Exercise is a widely used intervention for managing depression, but the specific molecular mechanisms underlying its antidepressant effect are unclear. In this narrative review, we aim to synthesize current knowledge on the molecular, neural, and physiological mechanisms through which exercise exerts its antidepressant effect and discuss the various exercise interventions used for managing depression. We conducted a narrative review of the literature on the topic of exercise and depression. Our review suggests that exercise impacts peripheral tryptophan metabolism, central inflammation, and brain-derived neurotrophic factors through the peroxisome proliferator-activated receptor γ activating factor 1α (PGC-1α) in skeletal muscles. The uncarboxylated osteocalcin facilitates “bone-brain crosstalk”, and exercise corrects atypical expression of brain-gut peptides, modulates cytokine production and neurotransmitter release, and regulates inflammatory pathways and microRNA expression. Aerobic exercise is recommended at frequencies of 3 to 5 times per week with medium to high intensity. Here we highlight the significant potential of exercise therapy in managing depression, supported by the molecular, neural, and physiological mechanisms underlying its antidepressant effect. Understanding the molecular pathways and neural mechanisms involved in exercise’s antidepressant effect opens new avenues for developing novel therapies for managing depression.

## 1. Introduction

Depression is a complex and multifaceted mood disorder that can profoundly impact individuals’ lives. It is characterized by persistent feelings of sadness, hopelessness, and a lack of motivation or interest in once-enjoyable activities [1,2,3]. Depression can significantly impair daily functioning, interfere with social relationships, and negatively impact physical health and quality of life. According to the World Health Organization, roughly 350 million individuals worldwide suffer from depression, representing a significant public health concern [4].

The etiology of depression remains elusive, with genetics, biology, and psychology among the factors implicated in its onset [5]. Additionally, studies have indicated that certain physical environmental factors, such as the presence of chemical pollutants in the air, may contribute to the incidence of depression in the populace [6].

To explicate the physiopathological basis of depression, the academic community has postulated an array of neurobiological hypotheses based on relevant experimental evidence and corresponding inferences. Such hypotheses include the Monoamine Neurotransmitter Hypothesis [7], Endogenous Opioid Peptide Hypothesis, and Neurotrophic Factor Hypothesis. Recent research indicates a correlation between depression and the inflammatory process. Some depressed patients exhibit elevated levels of pro-inflammatory cytokines in their blood, including interleukin-6 (IL-6), tumor necrosis factor-alpha (TNF-α), interleukin-1 beta (IL-1β), and C-reactive protein (CRP), when compared with control groups [8,9].

The Kynurenine (KYN) pathway is considered a potential mechanism connecting pro-inflammatory cytokines and depressive states [10,11]. Certain studies have revealed that the tryptophan (Trp)–tyrosine (KYN) metabolic system not only plays a pivotal role in the pathogenesis of diseases but also holds promise as a potential biomarker for environmental well-being [12,13]. The Trp-KYN metabolic system encompasses a series of endogenous bioactive metabolites derived from the indispensable amino acid tryptophan via the KYN metabolic pathway. Inflammatory mediators activate the KYN pathway, where tryptophan (TRP) serves as the precursor for serotonin, ultimately metabolizing into a compound known as kynurenine. Diminishing levels of TRP lead to a reduction in plasma serotonin concentrations, thereby amplifying the susceptibility to manifesting depressive symptoms [11].

Distinguished scholar Tanaka has notably emphasized the interplay between the Trp-KYN metabolic system and the immune system, as well as the conversion of tolerance to chronic low-grade inflammation. This exploration aims to unravel the intricate connections between chronic low-grade inflammation, KYN metabolites, and major psychiatric disorders, including depressive disorders [9,14,15]. Metabolizing through the KYN pathway gives rise to the production of quinolinic acid (QUIN) and 3-hydroxykynurenine (3-HK), which function as neurotoxic factors in the central nervous system (CNS) [10]. In addition to QUIN and 3-HK, other metabolites within the KYN pathway, such as kynurenic acid, which possesses neuroprotective/neurotoxic properties, are extensively investigated as potential biomarkers. The tryptophan-kynurenine metabolic system and its metabolites are considered potential targets for the treatment of cognitive impairment and headaches [16,17]. Furthermore, Tanaka delved into the impact of the tryptophan-kynurenine system on chronic pain as well as the central and peripheral sensitization of pain triggered by psychosocial and behavioral factors [14].

The advancements in neuroimaging and non-invasive brain stimulation (NIBS) techniques provide researchers with the opportunity to explore the underlying mechanisms of disorders such as depression [18]. By identifying the neural circuits and networks involved in these disorders, targeted interventions can be developed [19,20,21]. Through magnetic resonance imaging (MRI), structural changes have been observed in multiple brain regions in individuals with depression. Brain regions involved in emotion and cognitive functions are often impaired in depression, including the prefrontal cortex, temporal lobe, hippocampus, thalamus, striatum, and amygdala [22,23]. Specifically, dysfunction in the ventromedial prefrontal cortex (vmPFC), located in the lower frontal region, has been identified as one of the mechanisms underlying depressive disorders. However, recent research has emphasized the interaction between the autonomic nervous system and the anatomical and functional aspects of the prefrontal cortex, with network impairment playing a role in depression. To unravel the intricate pathophysiology of depression, researchers have created mathematical models that simulate specific neural and/or cognitive functions associated with depressive behavior [24].

The study of the treatment of depression heavily relies on preclinical models. Tanaka emphasizes that understanding the fundamental mechanisms of depressive disorders, identifying new therapeutic targets, and evaluating novel treatment approaches are all heavily dependent on preclinical models. However, current preclinical models have limitations that hinder their applicability to humans. Tanaka also highlights a significant challenge in neuroscience research, which is how to create preclinical models that accurately replicate the complexity of negative emotional disorders in the human body. He suggests the utilization of neuroimaging techniques such as magnetic resonance imaging (MRI) and positron emission tomography (PET), which allow researchers to visualize and quantify brain activity and structure in unprecedented detail, providing new avenues for studying depression [25].

Conventional treatments for depression typically involve medication and psychotherapy, which can be effective but are not universally accessible or tolerable [26,27,28,29,30,31,32]. A wealth of research indicates that exercise can improve symptoms of depression and promote overall physical and mental well-being [33,34,35,36,37,38]. Studies have shown that exercise can reduce the risk of developing depression and anxiety disorders [39,40,41,42,43,44].

Exercise can increase the production of certain neurotransmitters that regulate mood [45,46,47,48,49], reduce the release of the stress hormone cortisol, induce neurogenesis, and enhance the secretion of neurotrophic factors. For instance, brain-derived neurotrophic factor (BDNF), nerve growth factor (NGF), insulin-like growth factor-1 (IGF-1), and fibroblast growth factor-2 (FGF-2) have been found to improve neuronal survival, proliferation, and maturation [26,50,51,52]. In addition to BDNF, other neurotrophic factors, including glial cell line-derived neurotrophic factor (GDNF), and the neurotrophins NT-3 and NT-4, are also released in response to exercise [53,54,55,56].

In addition to its direct effects on the central nervous system, recent research suggests that exercise can modulate various neurobiological pathways [57,58,59] associated with depression, including muscle-brain [60,61], skeletal-brain, and gut-brain communication.

During exercise, the contraction of skeletal muscles increases the expression of peroxisome proliferator-activated receptor-gamma coactivator-1 alpha (PGC-1α), which in turn induces the expression of Fibronectin type III Domain-Containing protein 5 (FNDC5). This membrane protein is a precursor of irisin [62,63]. Irisin plays a beneficial role in promoting the expression of neurotrophic factors and other neuroprotective genes [64]. Similarly, during exercise, there is stimulation of the “bone-brain” crosstalk, which is a bone-derived endocrine-neural response system. After upregulation of ucOCN expression in response to exercise, it enters the bloodstream and activates the “bone-brain” crosstalk through mediating mechanisms such as the secretion of 5-HT/GABA, HPA axis function, inflammatory response, and the expression or signaling pathways of neurotrophic factors (like BDNF), GSK3β/β-catenin, TLR4/miR-223/NLRP3, thereby exerting its antidepressant effects [65,66,67,68,69,70]. The brain-gut axis is a bidirectional response system between the brain and the gut mediated by neuroendocrine pathways. Abnormal expression of brain-gut peptides in the periphery and central nervous system leads to depression, anxiety, gastrointestinal disorders, and metabolic diseases. Exercise can rectify the abnormal expression of peripheral or central brain-gut peptides (ghrelin, NPY, CCK, PYY, and GRP), thereby impacting the levels of monoamine neurotransmitters, HPA axis activity, the expression of neurotrophic factors and neuroplasticity, cell apoptosis, the metabolism of neurotoxic substances, and epigenetics, ultimately exerting its antidepressant effects [71,72,73,74].

Numerous studies have demonstrated the effects of exercise on modulating various pathways to improve depressive symptoms. These pathways may interact synergistically to promote healthy brain function and enhance mood [75,76,77,78]. The aim of this study is to analyze the neurobiological mechanisms underlying the relationship between exercise and depression, elucidate the potential mechanisms through which exercise improves depression, and evaluate the effectiveness of exercise interventions in treating depression. This will provide new theoretical foundations and research perspectives for the study of exercise and depression. Additionally, it will contribute to the development of more targeted exercise intervention programs for effectively managing depression.

## 2. Exercise-Induced Skeletal Muscle PGC-1α Expression and Improvement in Depressive Behavior

PGC-1α is a nuclear co-activator that activates downstream gene expression through its co-activating role with transcription factors. PGC-1α can bind to multiple nuclear receptors, such as the peroxisome proliferator-activated receptor-gamma (PPARγ), estrogen-related receptor alpha (ERRα), and participate in the transcription and post-transcriptional splicing modification of target genes [79]. PGC-1α is mainly expressed in tissues with high mitochondrial density and active oxidative metabolism, such as skeletal muscle, the liver, and the brain. Under physiological conditions, the expression level of PGC-1α is low, but it can be rapidly up-regulated when tissues are stimulated by external environmental cues, such as cold stimulation in adipose tissue or exercise in muscle tissue [80]. Downstream genes activated by PGC-1α mainly regulate mitochondrial biogenesis, oxidative metabolism, insulin sensitivity, and muscle fiber type conversion. The expression of neural PGC-1α may be associated with the pathogenesis of depression. Knockout of the PGC-1α gene affects dopaminergic neuron function and the expression of neuronal synapsin [81]. Overexpression of PGC-1α increases hippocampal BDNF expression and promotes hippocampal neurogenesis [82].

Skeletal muscle PGC-1α is an exercise-sensitive gene, and exercise can promote the expression of PGC-1α and regulate the activity of PGC-1α through multiple signaling pathways [83]. Skeletal muscle contraction stimulates the release of Ca^2+^ from the sarcoplasmic reticulum, activating calcineurin A (can) and calmodulin-dependent protein kinases (CaMKs), which promote the expression of PGC-1α. Exercise-induced generation of reactive oxygen species (ROS) in skeletal muscle increases the expression of PGC-1α through the AMPK and p38MAPK pathways. The mechanism by which exercise-induced skeletal muscle PGC-1α expression improves depressive behavior may be through regulating kynurenine (KYN) metabolism, peripheral inflammatory factors, and central brain-derived neurotrophic factor (BDNF) expression, thus indirectly exerting an antidepressant effect.

1. Exercise may balance skeletal muscle KYN metabolism through the PGC-1α/KAT pathway, alleviate the neurotoxic effects of KYN, and thereby exert an antidepressant effect. Aerobic exercise can increase the expression of skeletal muscle PGC-1α and KAT1-4 in healthy individuals [84]. Voluntary wheel running can promote the expression of skeletal muscle PGC-1α and KAT1, 3, 4, and increase the level of the KYN metabolite KYNA in the plasma of wild-type mice [84]. This suggests that the PGC-1α/KAT pathway activated by exercise regulates peripheral KYN metabolism.
2. Exercise may reduce the risk of depression induced by peripheral inflammation activation by activating skeletal muscle PGC-1α. The regulatory effect of exercise on peripheral inflammatory response may be related to the activation of skeletal muscle PGC-1α. Exercise can increase the expression of skeletal muscle PGC-1α, reduce peripheral inflammation levels, and thus alleviate the systemic inflammatory response [85]. Kohut et al. [86] found that exercise can improve stress-induced depressive behavior by reducing levels of interleukin (IL-6, IL-18) and TNFα. After exercise intervention, the plasma levels of IL-1β, IL-6, and TNFα in patients with depression were significantly reduced, and depressive symptoms were improved [87].
3. Exercise may exert an antidepressant effect by promoting the secretion of FNDC5/Irisin in skeletal muscle and increasing the expression of PGC-1α. Exercise-induced activation of PGC-1α can induce skeletal muscle to secrete FNDC5/Irisin [88]. Plasma Irisin levels in people who exercise regularly are significantly higher than those who lead sedentary lifestyles [89]. Wrann et al. [82] found that exercise can increase the expression of FNDC5 and BDNF in skeletal muscle and hippocampal tissue. When the FNDC5 gene in the liver of mice was activated, the level of BDNF in the hippocampal tissue also increased. This indicates that exercise can promote the secretion of FDNC5/Irisin in skeletal muscle through the activation of PGC-1α and that Irisin in the bloodstream can act as a remote secretion on brain tissue to exert a neuroprotective effect. However, there is still controversy surrounding Irisin research, as there are many sources of circulating Irisin in the blood, and it has not been proven yet whether the elevated circulating Irisin induced by exercise mainly originates from skeletal muscle, adipose tissue, or other tissues.

**Figure 1:**
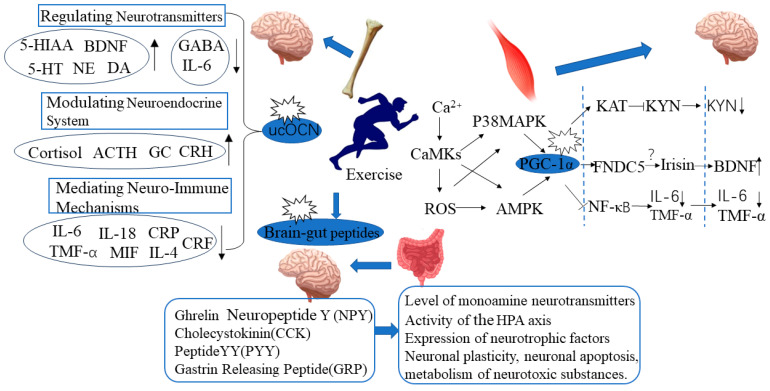
Mechanism of action of “exercise-brain crosstalk” in exercise’s anti-depressant effects. PGC-1α mediated peripheral-central “dialogue” involves upregulation of skeletal muscle PGC-1α by exercise, leading to a decrease in neurotoxic factors (such as KYN) released into the bloodstream from skeletal muscles; exercise-induced increase in skeletal muscle PGC-1α expression, which inhibits peripheral inflammation activation and reduces the action of peripheral pro-inflammatory cytokines on the brain; exercise-induced upregulation of skeletal muscle PGC-1α, which promotes the secretion of neuroprotective factors (such as Irisin) from skeletal muscles, thereby remotely activating BDNF secretion in the brain; Exercise promotes the expression of ucOCN in bones, which after crossing the blood-brain barrier, acts on various brain regions such as the hippocampus and prefrontal cortex, promoting the expression of neurotransmitters such as BDNF, DA, 5-HT, and NE, and inhibiting the expression of GABA. At the same time, exercise reduces the level of IL-6. ucOCN also activates the HPA axis by acting on brain regions such as the hypothalamus, promoting the expression of genes such as ACTH and CRH, and reducing the level of inflammatory factors through the neuroendocrine circulation, thus slowing down the neuroinflammatory response; Exercise can correct abnormal expression of peripheral or central brain-gut peptides (such as ghrelin, NPY, CCK, PYY, and GRP), thus influencing the levels of monoamine neurotransmitters, HPA axis activity, expression of neurotrophic factors, neuroplasticity, cell apoptosis, metabolism of neurotoxic substances, epigenetics, and other factors, exerting its antidepressant effects.

In summary, exercise-induced expression or activation of skeletal muscle PGC-1α has potential effects on regulating peripheral kynurenine metabolism, reducing the peripheral inflammatory response, and secreting neuroprotective factors. Through pathways such as alleviating neurotoxicity, reducing the central inflammation response, and promoting hippocampal neurogenesis, exercise exerts an antidepressant effect (Figure 1).

## 3. Exercise-Induced Expression of Bone-Derived Factor Ucocn Is Correlated with Improvement in Depressive Behavior

Uncarboxylated osteocalcin (ucOCN) is a hormone secreted by the bone that has diverse regulatory effects on organs, tissues, and metabolism. ucOCN can also exert antidepressant effects via neural pathways. A study showed that the levels of ucOCN were significantly decreased in patients with depression and negatively correlated with the severity of depression, suggesting that ucOCN may be a biological marker for depression. In addition, drugs such as SSRIs and lithium can modulate the expression of the ucOCN gene. A recent study indicated that treatment with SSRIs can result in a decrease in the expression of the ucOCN receptor Gprc6a and a reduction in the biological activity of ucOCN, which may impact the therapeutic effect of treating depression. This highlights the importance of ucOCN as a potential target for depression treatment [90].

The bone-derived factor ucOCN can mediate monoamine neurotransmitter, neuroendocrine, neuroimmune, neural regeneration, and gene expression and thus regulate the occurrence and improvement of depression in brain regions such as the CA3 area of the hippocampus and the cingulate gyrus via crossing the blood-brain barrier. As a mechanical stimulus-sensitive gene derived from the bone, ucOCN enters the bloodstream upon its expression being upregulated by exercise and promotes the secretion of 5-HT/GABA, enhances HPA axis function, alleviates inflammatory responses, promotes the expression of neurotrophic factors such as BDNF, and achieves a “brain-bone connection”, exerting anti-depressive effects.

Exercise can increase the secretion of monoamine neurotransmitters [91]. Studies have found that the levels of monoamine neurotransmitters (such as NE, DA, 5-HT, and 5-HIAA) in different brain regions of rats with chronic mild unpredictable stress-induced depression were significantly decreased, whereas long-term swimming training improved depressive behaviors while restoring and increasing the secretion of monoamine neurotransmitters [92]. Exercise upregulates the expression of ucOCN in the bone tissue of depressed rats, thereby promoting the secretion of 5-HT and DA in the serum and improving their depressive-like behavior [92]. In addition, 8 weeks of treadmill training upregulated ucOCN expression in the bones of chronic unpredictable mild stress (CUMS) rats and, after entering the bloodstream, promoted 5-HT secretion while inhibiting that of GABA, improving their depressive-like behavior [93]. Furthermore, 2 months of treadmill training (30 min/day) significantly increased ucOCN levels in the serum of T2DM mice with comorbid depression, improving their depressive-like behavior [94]. These effects are closely related to the activation of ucOCN by exercise and its mediation of the Gpr158/BDNF pathway, which promotes the secretion of neurotransmitters and regulates the occurrence of cognitive disorders such as depression. These findings suggest that the secretion of monoamine neurotransmitters mediated by ucOCN realizes the “bone-brain crosstalk”, thereby mediating the anti-depressive effects of exercise. In addition, a study of 80 obese adolescents who exercised for 3 months to lose weight found that serum ucOCN levels were significantly positively correlated with urinary cortisol levels and improvements in depressive-like behavior [95].

Exercise can upregulate ucOCN expression by increasing osteoblast (OB) activity, thereby inhibiting the mRNA expression of inflammatory factors such as IL-6, IL-18, and TNF-α, and significantly improving depressive-like behavior [96]. This effect is related to the activation of ERK and STAT pathways after upregulation of ucOCN expression, which downregulates the mRNA and protein expression of IL-6 and IL-8 in the hippocampus and upregulates the expression of VGF and BDNF through the MDA/SOD/Nrf2/HO1 pathway [97]. University students with depressive symptoms showed upregulated ucOCN expression and a significant negative correlation with downregulated TNF-α and CRP in the serum after 8 weeks of aerobic exercise, and their depressive behavior was significantly improved [98]. These studies reveal that ucOCN mediates the “bone-brain crosstalk” by regulating the neuroimmune system in brain tissue, thus mediating the anti-depressive effects of exercise. Through the mediation of the neuroimmune system, ucOCN achieves the “bone-brain crosstalk” and exerts its anti-depressive effects through exercise (Figure 1).

## 4. Exercise-Mediated Brain-Gut Peptide Expression and Improvement in Depressive Behavior

Exercise can regulate the expression of central/peripheral gastrointestinal peptides such as ghrelin, neuropeptide Y (NPY), cholecystokinin (CCK), etc. Research has shown that exercise can regulate depressive behavior by modulating the ghrelin/GHS-R1a pathway. Two weeks of voluntary exercise can significantly upregulate GHS-R protein expression in the hippocampus and hypothalamus [99]. Studies have shown that GHS-R knockout mice have decreased cell proliferation and survival in the hippocampus, increased apoptosis of dentate gyrus cells, and more severe depressive behavior after CSDS; P7C3, a neuroprotective aminopropyl carbazole, can enhance neurogenesis and exert an antidepressant effect in GHS-R knockout mice [100]. It has been reported that P7C3 can simulate the effect of exercise on enhancing hippocampal neurogenesis. These findings suggest that exercise can prevent depressive behavior at least in part by inducing hippocampal neurogenesis through stimulation of the ghrelin/GHS-R signaling pathway. Liu et al. [101] found that in a CUMS model of depression in rats, ghrelin and its receptor expression in the prefrontal cortex increased, accompanied by increased GSK-3β activity and NLRP3 expression; 4 weeks of moderate-intensity swimming exercise reversed these changes, suggesting that exercise may improve depressive behavior by regulating ghrelin and its receptor, GSK-3β activity, and NLRP3. In addition, ghrelin administration in the rat hippocampus dose-dependently increased the activity of nitric oxide synthase (NOS) in the dentate gyrus and thereby increased the synthesis of nitric oxide (NO), promoting long-term potentiation (LTP) in the hippocampus [102]; however, NOS activity is significantly different between exercised and unexercised animals. In a depression model with corticosterone administration and high-fat diet feeding, the antidepressant effects of the exercise-mimicking drug AICAR require increased endothelial NOS activity and NO production [103]. Therefore, it is speculated that the activation of NOS by ghrelin to increase NO synthesis and enhance synaptic plasticity is an important mechanism underlying the antidepressant effects of exercise. Current research on exercise regulation of ghrelin expression is mainly focused on metabolic diseases, with blood samples being the main type of detection. Therefore, much more work needs to be done on the changes in ghrelin expression in different brain regions regulated by exercise.

Research has shown that NPY mediates the antidepressant effects of exercise. Voluntary running or the combined use of antidepressants and voluntary running can upregulate NPY and Y1R expression in the hippocampus of FSL rats, accompanied by increased proliferation of new hippocampal cells and improved depressive behavior. Hippocampal NPY levels were positively correlated with the number of BrdU-immunopositive cells [104], suggesting that exercise may promote hippocampal cell proliferation and neurogenesis through activation of NPY/Y1R, thereby exerting antidepressant effects. Swimming exercise reversed the decreased levels of NPY, BDNF, and VGF mRNA in the hippocampus of CUMS-induced rats and restored the expression of the anti-apoptotic protein Bcl-xl and the pro-apoptotic protein Bax to near-normal levels, indicating that the antidepressant effects of exercise are related to the regulation of neurotrophic factors and anti-apoptotic mechanisms by NPY [105]. In addition, exercise activates epigenetic reprogramming of the NPY gene to exert its antidepressant effects. Specifically, exercise increases NPY mRNA levels in the hippocampus of FSL rats and downregulates Hdac5 mRNA levels, resulting in an increase in H3K18 acetylation levels [106]. It is known that EP300 is the main histone acetyltransferase required to maintain H3K18ac levels in the body, and EP300 can regulate cyclooxygenase-2 (COX-2). Abnormal COX-2 function can cause inflammation and is considered one of the targets in depression research. Based on the important role of NPY receptors in the pathogenesis of depression, future research needs to explore the effects of acute or long-term exercise on NPY receptor subtypes in depression-related brain areas.

Exercise can serve as an independent regulatory factor affecting peripheral CCK metabolism. Acute exercise can sustainably increase plasma CCK levels, which is associated with an increased ratio of free tryptophan to branch-chain amino acids in the blood [107]. In contrast, although four weeks of exercise slightly decreased plasma CCK levels, the change was not significant, suggesting that peripheral CCK metabolism may be more sensitive to acute exercise than chronic exercise or that four weeks of exercise may not be enough to change peripheral CCK levels. After acute intense exercise, the release of intestinal hormones (gastrin, CCK, and pancreatic polypeptide) and stress hormones (norepinephrine, cortisol, and growth hormone) immediately increases, and this increase is independent of the feeding effect [108]. Acute treadmill exercise can alleviate panic symptoms induced by CCK-4 injection [109]. Overall, the current research on the effect of exercise on CCK expression focuses mostly on its regulation of appetite and is mainly centered on the peripheral system. The role of CCK in exercise-induced antidepressant effects is largely unknown (Figure 1).

## 5. The Relationship between Exercise and Mental Health: The Role of Inflammatory Factors and Neurotransmitter Changes

In recent years, there has been extensive research on the relationship between exercise and mental health. The discussion includes the relationship between inflammatory factors and tyrosine metabolism products, neurotransmitter changes, neuroimaging evidence, and the manifestation of psychoneurological symptoms to comprehensively explore the role of exercise in mental health [18,25].

Inflammatory factors play an important role in mental health. Some studies have shown that emotional disorders such as depression and anxiety are associated with increased levels of inflammatory factors [110]. Preclinical model studies have found that inflammatory factors can induce negative emotional behavior and affect the normal function of neurotransmitters. In addition, tyrosine metabolism products are also considered to be related to mental health because they are involved in the synthesis and stabilization of neurotransmitters.

Neurotransmitters are chemical messengers in the nervous system that play an important role in emotion and cognitive function [5]. Research shows that exercise can alter the release and reuptake of neurotransmitters, thereby regulating mood and psychological states. For example, exercise can increase the release of serotonin and dopamine, which are associated with positive emotions and feelings of happiness. At the same time, exercise can also decrease the release of glutamate, which is associated with anxiety and depression.

Neuroimaging studies have also provided insights into the effects of exercise on mental health. Some studies have found that people who engage in long-term physical activity show positive changes in brain structure and function. For example, exercise can increase the volume of the hippocampus, which is associated with cognitive function and emotional regulation. In addition, exercise can increase the activity of the prefrontal cortex, which is associated with decision-making and emotional regulation [111].

Psychoneurological symptoms are common manifestations of mental health problems. Some studies have found that exercise can alleviate symptoms such as anxiety, depression, and stress. This may be because exercise can enhance an individual’s psychological resilience and self-esteem and promote social interaction while reducing feelings of loneliness.

Overall, research results indicate that exercise has a positive impact on mental health. By regulating changes in inflammatory factors, neurotransmitters, and the effects on brain structure and function, exercise can improve emotions, alleviate anxiety and depression symptoms, and enhance an individual’s mental health. Therefore, incorporating exercise into daily life and maintaining moderate physical activity is one of the most important strategies for promoting mental health. While the benefits of exercise for mental health have been widely recognized, it is still necessary to choose appropriate exercise methods based on individual physical conditions and interests and gradually increase the intensity and duration of exercise for optimal results. At the same time, it is recommended to consult professional doctors or health experts’ advice before starting any exercise plans to ensure safety and effectiveness.

## 6. Effects of Different Exercise Intervention Programs on Improving Depression

### 6.1. Effects of Different Types of Exercise on Improving Depression

#### 6.1.1. Aerobic Exercise

Aerobic exercise can effectively reduce the average score of depression rating scales in patients with depression, and the good effect can still be maintained after 12 months [112]. After 4 weeks of cognitive training with a combination of aerobic exercise and mental relaxation training, the research found a decrease in anxiety levels and an improvement in subjective quality of life in patients with depression [113]. After 8 weeks of moderate-intensity aerobic exercise intervention, depression symptoms, rumination, and cognitive control improved significantly in patients with depression [114]. A meta-analysis of the effects of an aerobic exercise intervention on postpartum depression found that group aerobic exercise, individual aerobic exercise, and combined exercise can effectively reduce postpartum depression [115].

#### 6.1.2. Yoga

Meta-analysis and systematic review studies suggest that yoga may reduce symptoms of depression [116]. Studies have shown that yoga exercise is as effective as antidepressant medication in treating major depressive disorder (MDD) [117]. James-Palmer et al. [118] reviewed 27 studies on different health conditions of young people to investigate the intervention effects of yoga on anxiety and depression syndrome. They found that 70% of studies showed significant improvements, 58% showed reduced symptoms of anxiety and depression, 25% showed only reduced anxiety, and 70% of studies that evaluated anxiety alone showed improvement with exercise. Furthermore, 40% of studies that evaluated depression only showed positive intervention effects. Another study confirmed that mindfulness yoga programs are more effective than stretching and resistance training programs in reducing anxiety and depression symptoms and improving mental health and quality of life in patients with mild to moderate Parkinson’s disease [119].

#### 6.1.3. Resistance Training

Gordon et al. [120] conducted a meta-analysis on the correlation between resistance training and depression and found that resistance training significantly reduced depression symptoms in adults, and there was no significant correlation between the prescription of resistance training, participant health status, strength improvement, and antidepressant effects. After 12 weeks of resistance training in post-ischemic stroke depression patients, resistance training significantly reduced depression scores compared with the control group, and all physical functions improved effectively. Studies have shown that there is a negative correlation between strength improvement and depression level, and strength training is beneficial for reducing depression severity [121]. Studies found that 9 months of resistance training improved the quality of life and reduced depression levels in elderly depressed patients [122]. The psychological regulation effect of resistance training was more significant than that of aerobic training twice a week. One study has confirmed that both resistance training and endurance training can improve mood, but only endurance training can additionally reduce anxiety [123]. After exercise intervention in elderly depressed patients clinically diagnosed and receiving regular treatment, Studies evaluated the intervention effect using the Hamilton Depression Scale and Beck Depression Scale and showed that resistance training significantly reduced the depression level of the subjects [124]. After 24 weeks of strength training, compared with the control group, the neurotransmitters 5-hydroxytryptamine (5-HT), dopamine (DA), adrenaline, and noradrenaline (NE or NA) in elderly female depressed patients were significantly reduced, while the depression factor did not change significantly [125]. Kang et al. found that 8 weeks of aerobic and resistance training effectively improved depression behavior, neuronal damage, and synaptic plasticity reduction induced by chronic unpredictable mild stress (CUMS), but the mechanisms were different: aerobic exercise intervened in depression through PGC-1α/ERRα/FDNC5, while resistance training intervened through activation of IGF-1/IGF-1R/Akt/mTOR [126]. Therefore, aerobic exercise may be a more effective intervention in depression exercise therapy (Table 1). Although resistance training has also shown positive preventive and therapeutic effects on depression in some studies, there is still some controversy, and further research is needed to confirm.

#### 6.1.4. Whole-Body Vibration

In recent years, whole-body vibration has played an important role in improving health and psychological well-being as a widely accepted form of passive exercise. Studies have shown that whole-body vibration also has a positive effect on depression, improving both the psychological and physiological functions of patients.

Whole-body vibration can enhance muscle contraction and relaxation, thereby improving metabolism and nervous system function. In addition, it can also enhance neuron growth and protect neurons from damage, which in turn improves brain function. Whole-body vibration also increases the expression of FNDC5 and BDNF, which are beneficial proteins for the body that increase muscle strength and protect the nervous system, and play a positive role in treating depression [127,128].

Recent research has shown that whole-body vibration can be used as a simple and effective new treatment method for depression [129]. Some trials have found that whole-body vibration significantly improves depression symptoms such as emotional depression, negativity, lack of expression, and physical fatigue, thereby improving the quality of life and physical health of patients. Therefore, using whole-body vibration as a non-invasive new treatment method for depression has broad application prospects.

### 6.2. Effects of Different Intensity, Frequency, and Volume of Exercise on Depression

#### 6.2.1. The Impact of Exercise Intensity on Treating Depression

In one study [130], the effects of moderate-continuous versus high-intensity interval training on depression levels and inflammatory factors were investigated in a group of 61 university students with depression. The results showed that moderate-continuous training led to a significant reduction in depression symptoms and tumor necrosis factor-alpha (TNF-α) levels, whereas high-intensity interval training reduced depression symptoms but increased perceived stress and inflammatory factors such as TNF-α and interleukin-6 (IL-6). Another investigation [131] involving male patients with moderate depression randomly assigned participants to three groups with interventions of varying intensities over 6 weeks. The study found that moderate and high-intensity aerobic exercise led to significantly improved depression levels, while low-intensity exercise had less noticeable effects. The findings suggest that moderate and high-intensity aerobic exercise could be more effective treatments for depression compared with low-intensity exercise alone.

#### 6.2.2. The Influence of Exercise Frequency on Treating Depression

A study assessing the correlation between exercise frequency and depression in Australian perinatal women found that decreased exercise frequency during the perinatal period was associated with increased depression and anxiety symptoms [132]. In a study [122] involving elderly patients with depression who underwent a 3-month resistance training program, participants were randomly assigned to four groups: RT1, RT2, RT3, and a non-training control group. The training group underwent six months of resistance training once, twice, or three times per week, respectively. The researchers evaluated the participants’ quality-of-life and depression symptoms after training and found that during the period between 3 and 9 months post-training, the RT1 group showed a greater decline in quality-of-life compared with the RT2 and RT3 groups. However, the RT2 group exhibited a better quality of life compared with the other two groups. In a small randomized controlled trial with 23 participants, Legrand et al. [133] found that patients who engaged in exercise 3–5 times per week for 30 min each session showed a greater reduction in Beck Depression Inventory scores compared with those who exercised only once a week. Cramer et al. [116] discovered that exercising at least twice a week could help prevent elderly depression after analyzing data from 1142 participants for 2 years. Moreover, Kanamori et al. [134] found that people who exercised at least twice a week had a significantly lower risk of developing depression compared with those who did not exercise after tracking the participants for two years. A comprehensive analysis of regular exercise and nutrient intake found that only exercise was significantly positively associated with a decrease in depression. Notably, the intervention effect of exercising 3–4 times per week was better than exercising 1–2 times per week and more than 5 times per week [135]. Despite the insufficient research focusing on the influence of single-factor exercise frequency on depression, the results have been consistent in showing that higher exercise frequency is more beneficial for preventing and treating depression.

#### 6.2.3. The Influence of Exercise Volume on Treating Depression

To investigate the existence of a dose-dependent relationship between physical activity intervention and depression, researchers searched and analyzed multiple databases and found that a large amount of occupational or leisure-time physical activity was usually associated with a reduction in depression symptoms [136]. Two randomized controlled trials showed that high-intensity aerobic exercise or resistance training that consumed at least 17.5 kcal/kg per week was more effective in reducing depression symptoms than low-intensity exercise that consumed 7 kcal/kg or less per week [133,137]. A study found that when the exercise volume was maintained at a level of 1200–3000 METs-min/week, the risk of depression was significantly reduced. The risk of depression symptoms decreased by 10% when the exercise volume ranged from 1200–1800 METs-min/week and decreased by 14% when the exercise volume ranged from 1800–3000 METs-min/week. When the exercise volume was greater than 3000 METs-min/week, there was no significant effect on reducing depression [138]. To investigate the effect of exercise volume on depression intervention, researchers set four different doses of aerobic exercise that ensured at least moderate intensity for community-dwelling elderly patients with depression: (1) 3 times/week, 15 min/time; (2) 3 times/week, 30 min/time; (3) 6 times/week, 15 min/time; (4) 6 times/week, 30 min/time. The results showed that even the smallest volume of aerobic exercise (i.e., 3 times a week for 15 min each time) could significantly reduce depression symptoms and have a comparable effect with other exercise volumes [139]. A study found that if all participants engaged in at least 1 h of physical activity per week, the incidence of future depression could be reduced by 12% [140]. Moderate physical activity is beneficial for reducing the risk of depression in women, while high exercise volumes increase the risk of depression in men. The research shows that women have a lower probability of developing depression if they engage in 150–299 min of moderate-intensity physical activity per week, while a higher risk of depression is associated with engaging in 300 min or more of intense physical activity per week or performing 2250 metabolic equivalents of moderate-intensity physical activity. The relationship between depression and physical activity is largely dependent on exercise intensity and gender [141]. Similarly, researchers found that gender and a family history of depression are also factors influencing the choice of the physical activity intervention dose for depression. Men and women without a history of major depressive disorder (MDD) have a higher remission rate with high-dose exercise, while women with a history of MDD have a higher remission rate with low-dose exercise [142]. The relationship between exercise volume and depression intervention effects is still somewhat controversial, but from existing research, it appears that good anti-depressive effects can be achieved with a low volume of exercise, and higher exercise volumes may bring greater benefits.

## 7. Discussion

In this review, we have explored the advancements in neurobiology and the effects of exercise on depression. Research findings suggest that exercise can serve as a promising intervention for combating depression and may hold potential benefits for mental health. The etiology of depression remains unclear, involving factors such as genetics, biology, and psychology. Neurobiological hypotheses indicate a link between depression and inflammatory processes, with the kynurenine pathway being a potential mechanism. The tryptophan-kynurenine metabolism system and its metabolites present potential targets for treating cognitive impairment, headaches, and chronic pain. Through a retrospective examination of the molecular pathways and neurobiological mechanisms underlying the antidepressant effects of exercise, several exercise-induced factors have been identified, including neurotrophic factors, PGC-1α activated by muscle contraction-induced reactive oxygen species, exercise-induced ucOCN produced by skeletal muscle, and exercise-induced improvement in aberrant brain-gut peptide expression. These factors play crucial roles in alleviating depressive behaviors. Furthermore, this review retrospectively investigates the impact of different exercise intervention strategies on alleviating depression symptoms, such as aerobic exercise, yoga, resistance training, and whole-body vibration. Various physical activities have shown varying degrees of symptom reduction in depression. Moderate to high-intensity exercise demonstrates more favorable effects on improving depression, and a frequency of 3–4 times per week yields better results in alleviating depressive symptoms. The minimum exercise volume required to alleviate depression symptoms may differ among individuals, and higher exercise volumes may yield greater benefits.

Integrative biology posits that exercise challenges the homeostasis of the internal environment in the human body, disrupting the preexisting balance of cells, tissues, and organs and prompting them to respond. This multi-level integration and response establish a new dynamic equilibrium in the body, enhancing muscle energy and oxygen supply to meet the demands of muscle contraction [143,144,145]. The disturbance and reshaping of this systemic homeostasis not only benefit the skeletal muscles themselves but also have positive effects on multiple organs and systems in the body [146,147].

Recent research has found that the exercise-sensitive gene PGC-1α in skeletal muscles regulates peripheral metabolism, which, in turn, affects brain health. Exercise increases the expression of the skeletal muscle-derived gene ucOCN, which enters the bloodstream. This gene plays a role in achieving “bone-brain connectivity” by influencing various processes such as the secretion of 5-HT/GABA, HPA axis function, inflammatory response, and expression of neurotrophic factors such as BDNF. By activating signaling pathways like GSK3β/β-catenin and TLR4/miR-223/NLRP3, exercise exerts antidepressant effects. The brain-gut axis, a responsive system mediated by neuroendocrine pathways, is crucial for human health and behavior. Abnormal expression of brain-gut peptides, like ghrelin, NPY, CCK, PYY, and GRP, in the peripheral and central systems can lead to depression, anxiety, gastrointestinal diseases, and metabolic disorders. Exercise corrects the abnormal expression of these peptides, impacting neurotransmitter levels, HPA axis activity, neurotrophic factor expression, neuroplasticity, apoptosis, metabolism, and epigenetics, thereby exerting antidepressant effects.

Further research is needed to deepen our understanding of how exercise promotes “periphery-brain” crosstalk. The mechanisms by which skeletal muscle secretions and peripheral inflammatory factors act on brain tissue are still unclear. While there are numerous skeletal muscle secretions, only a few have been identified as having an impact on brain tissue. Future studies should focus on screening small molecules that can cross the blood-brain barrier or exploring corresponding receptors on the blood-brain barrier. Additionally, the discovery of the meningeal lymphatic system opens up avenues for further research to determine the mechanisms by which peripheral inflammation factors induce central inflammation. This is of significant importance in establishing the connection between peripheral inflammation and depression as well as exploring the inflammatory mechanisms underlying the antidepressant effects of exercise. Exercise activates various genes in skeletal muscles, but only a few have been identified as playing a role in mediating muscle-brain crosstalk. Delving deeper into these issues will allow for a more comprehensive understanding of the integrative biology principles underlying the beneficial effects of exercise on brain health. There is a greater diversity of skeletal muscle secretions and specific expressed factors, but currently, only the role of ucOCN in exercise-induced antidepressant effects has been confirmed. Future studies should focus on further screening for bone secretions or specific small molecules that may penetrate the blood-brain barrier and exert effects on brain tissue. Likewise, exploring the corresponding receptors on the blood-brain barrier and studying their mechanisms will be valuable. The impact of different exercise modalities, intensities, and durations on brain-gut peptide levels in individuals with depression and animal models is not yet fully understood.

Further investigations grounded in the principles of integrative biology have revealed the existence of a “periphery-brain” crosstalk during the process of exercise, shedding new light on the role of physical activity in improving mental well-being. Not only does exercise lead to favorable adaptations within the central nervous system, but it also establishes molecular connections between peripheral organs and brain adaptations. These findings provide a neurobiological foundation for understanding how exercise can ameliorate psychological health conditions such as depression and anxiety, offering a fresh perspective and support for the study of the mechanisms underlying the beneficial effects of exercise on mental disorders. Moreover, they present novel evidence for the beneficial impacts of exercise on overall health. Knowledge of the mechanisms of “periphery-brain” crosstalk during exercise opens up new avenues for researchers to explore alternative approaches for treating mental health conditions like depression. In the future, scientific paradigms will increasingly rely on a “multi-factorial network” framework to elucidate the mechanisms through which exercise exerts its antidepressant effects.

This review also offers novel insights into the role of specific modes and intensities of exercise in alleviating depressive symptoms, providing valuable information for future investigations. However, the consideration of exercise interventions as clinical treatments for mental disorders such as depression requires more rigorous randomized controlled trials to evaluate the efficacy of exercise interventions in treating depression [148,149,150,151]. While the effects of different types, intensities, frequencies, and durations of exercise on depression have been studied, further research is needed to determine the optimal exercise regimen for individuals with depression. Additionally, exploring factors that influence exercise adherence and strategies for maintaining long-term engagement in physical activity are essential.

Moreover, the advancements in neuroimaging and non-invasive brain stimulation techniques [25,152,153], along with the interdisciplinary research involving artificial intelligence [154,155,156], offer new avenues for achieving precision medicine and tailored exercise interventions in the clinical research and treatment of mental disorders such as depression. The study of depression treatment heavily relies on preclinical models. Tanaka emphasizes that understanding the fundamental mechanisms, identifying therapeutic targets, and evaluating new treatments all depend on these models. However, current preclinical models have limitations that hinder their applicability to humans. Tanaka highlights the challenge of replicating the complexity of human emotional disorders in preclinical models. He suggests using neuroimaging techniques like MRI and PET to visualize and quantify brain activity and structure in unprecedented detail, providing new avenues for studying depression [25].

The significance of exercise therapy in treating depression lies in its non-pharmacological self-management approach, which can be used in conjunction with other treatment modalities such as medication and psychotherapy to offer comprehensive depression management. It is a cost-effective and easily accessible treatment option with minimal side effects. Furthermore, exercise provides a positive and beneficial daily activity, offering patients an opportunity for hope and an improved quality of life.

As a narrative review, our study extensively reviewed numerous studies and literature, delving into the neurobiological mechanisms and intervention effects of exercise on depression. However, there are also limitations. We relied solely on published studies, which may introduce publication bias and influence our research findings. The heterogeneity of research methods makes it difficult to generalize the results and establish causal relationships between exercise and the alleviation of depressive symptoms. Therefore, our review should be considered a starting point for future research rather than a definitive conclusion.

## 8. Conclusions

By conducting a retrospective analysis of the current literature and employing an integrative biological approach, this study explores the neurobiological mechanisms through which exercise may improve depression. It specifically focuses on the ‘periphery-brain cross talk’ mechanism and provides an overview of the intervention effects of different exercise programs. Specifically, exercise can regulate the production and release of neurotransmitters, hormones, and inflammatory factors. Through its influence on the function and secretions of peripheral organs, exercise can modulate the activity and function of brain circuits. This “periphery-brain” communication may play a crucial role in the process of exercise-induced improvement of depression. Further research should focus on uncovering exercise-sensitive genes related to depression within peripheral organs and revealing their associations with depression. In addition, the use of a “multi-factor network” interpretive approach is an important direction for investigating the mechanisms of exercise as an anti-depressant. By comprehensively analyzing the effects of exercise on neurotransmitters, hormones, and inflammatory factors, we can establish a comprehensive model to explain the anti-depressant effects of exercise. To achieve precise exercise interventions for depression, future research should focus on rigorous randomized controlled trials, ensuring the obtained results are highly reliable and reproducible. Additionally, the use of interdisciplinary technologies such as neuroimaging and artificial intelligence algorithms can enhance our understanding of the impact of exercise on brain structure and function, enabling the identification of tailored exercise intervention strategies for individuals and improving the individualization and efficacy of treatment.

## Figures and Tables

**Table 1:** The effects of different types of exercise interventions on depression.

Author, Year	Exercise	Exercise Prescription	Effectiveness
Dilorenzo et al., 1999 [112]	Cycling	70–80%HRR, 4 times a week, 12 weeks.	Beck Depression Inventory Scores show a significant reduction, intervention remains effective one year later.
Olson et al., 2017 [114]	Running	40–65%HRR, 3 times a week, 8 weeks.	Improvement in cognitive control, depressive symptoms, and ruminative thinking patterns in patients with depression.
Dziubek et al., 2016 [123]	Endurance exercise	3 times a week, 24 weeks.	Enhancing mood and reducing anxiety.
Kwok et al., 2019 [119]	Yoga	90 min per session, 8 weeks.	Reducing anxiety and depression symptoms in Parkinson’s patients.
James-Paler et al., 2020 [118]	Yoga	30 min per session, 2–3 times per week, 12 weeks.	Reducing anxiety and depression symptoms in teenagers.
Gordon et al., 2018 [120]	Resistance training	3 times per week, 52 weeks.	Significantly reducing symptoms of depression in adults.
Kim et al., 2019 [125]	Resistance training	30–60 min per session, 3 times a week, 24 weeks.	There is a significant decrease in neurotransmitters 5-HT, DA, NA, and NE in elderly female patients with depression.
Moraes et al., 2020 [124]	Resistance training	2 times per week, 12 weeks.	Significantly reduces HAMD and BDI scores in elderly patients with depression.

## Data Availability

No applicable.
