# Correction: Ren et al. Exercise for Mental Well-Being: Exploring Neurobiological Advances and Intervention Effects in Depression. *Life* 2023, *13*, 1505

**DOI:** 10.3390/life14070816

**Published:** 2024-06-27

**Authors:** Jianchang Ren, Haili Xiao

**Affiliations:** Institute of Sport and Health, Guangdong Provincial Kay Laboratory of Development and Education for Special Needs Children, Lingnan Normal University, Zhanjiang 524037, China; xiaohl@lingnan.edu.cn

There was an error in the original publication [1]. A correction has been made to Section 3 “Exercise-Induced Expression of Bone-Derived Factor Ucocn Is Correlated with Improvement in Depressive Behavior”:

Original: In addition, a study of 80 obese adolescents who exercised for 3 months to lose weight found that serum ucOCN levels were significantly positively correlated with urinary cortisol levels and improvements in depressive-like behavior [95].

Corrected: In addition, a study demonstrated that exercise helps improve anxiety behavior induced by VCD in menopausal mice, promotes neurogenesis of granule cells in the hippocampus, and inhibits apoptosis of hippocampal cells. These effects are associated with the increased circulation of osteocalcin induced by exercise [95].

Original: University students with depressive symptoms showed upregulated ucOCN expression and a significant negative correlation with downregulated TNF-α and CRP in the serum after 8 weeks of aerobic exercise, and their depressive behavior was significantly improved [98].

Corrected: High-impact exercise can significantly increase serum osteocalcin levels in postmenopausal women. Additionally, high-impact training programs can improve functional mobility and health-related quality of life in postmenopausal women [98].

The references:

Original reference [95]: Okbay, G.A.; Alikasifoglu, M.; Sen, D.E.; Erginöz, E.; Demir, T.; Kucur, M.; Ercan, O. The Relationship of Disordered Eating Attitudes with Stress Level, Bone Turnover Markers, and Bone Mineral Density in Obese Adolescents. *J. Clin. Res. Pediatr. Endocrinol.* **2017**, *9*, 237–245.

Corrected to [95]: Kang, Y.; Yao, J.; Gao, X.; Zhong, H.; Song, Y.; Di, X.; Feng, Z.; Xie, L.; Zhang, J. Exercise ameliorates anxious behavior and promotes neuroprotection through osteocalcin in VCD-induced menopausal mice. *CNS Neurosci. Ther.* **2023**, *29*, 3980–3994. https://doi.org/10.1111/cns.14324.

Original reference [98]: Huang, T.H.; Lin, J.C.; Ma, M.C.; Yu, T.; Chen, T.C. Acute responses of bone specific and related markers to maximal eccen-tric exercise of the knee extensors and flexors in young men. *J. Musculoskel. Neuron.* **2020**, *20*, 206–215.

Corrected to [98]: Sen, E.I.; Esmaeilzadeh, S.; Eskiyurt, N. Effects of whole-body vibration and high impact exercises on the bone metabolism and functional mobility in postmenopausal women. *J. Bone Miner Metab.* **2020**, *38*, 392–404. https://doi.org/10.1007/s00774-019-01072-2.

The authors state that the scientific conclusions are unaffected. This correction was approved by the Academic Editor. The original publication has also been updated.
